# An Unusual Presentation of Extremely Early Neonatal Cirrhosis in Shwachman-Diamond Syndrome: A Case Report

**DOI:** 10.7759/cureus.38583

**Published:** 2023-05-05

**Authors:** Tejaswy Reddy, Rakesh Kotha, Alimelu M

**Affiliations:** 1 Department of Neonatology, Niloufer Hospital, Hyderabad, IND; 2 Department of Neonatology, Osmania Medical College, Hyderabad, IND

**Keywords:** fulminant liver failure. hepatic encephalopathy, bone, pancytopenia, marrow failure syndrome, ascites

## Abstract

Exocrine pancreatic insufficiency, haematological dysfunction, and skeletal abnormalities are the three clinical characteristics of the rare inherited bone marrow failure syndrome (IBMFS), known as Shwachman-Diamond syndrome (SDS). Cirrhosis at a neonatal age is uncommon and is typically not documented, as in neonatal presentation. Here, we present a case of SDS in which bi-cytopenia with macro-nodular cirrhosis emerged before the age of one month. Utilising genetic testing on the infant and both parents, we were able to confirm the diagnosis. We were expecting a higher-level liver transplant set-up, but the infant passed away in the interim. Genetic studies play a significant part in the diagnosis of difficult cases.

## Introduction

Shwachman-Diamond syndrome (SDS), a rare inherited bone marrow failure syndrome (IBMFS), is an autosomal recessive disorder with clinical features that include a triad of exocrine pancreatic insufficiency, haematological dysfunction and skeletal abnormalities. It is the second most common cause of pancreatic insufficiency after cystic fibrosis [1]. The most common age of presentation is infancy, with a higher incidence in males. A mutation in the Shwachman-Bodian-Diamond syndrome (SBDS) gene on chromosome 7 is found in 90% of the cases [2,3]. However, other varied presentations of SDS include cardiac abnormalities, immune dysfunction, life-threatening infections, neurobehavioural impairment and hepatic dysfunction. Thus, the phenotypic spectrum is varied and might also include more subtle and clinically inapparent findings [4]. Neonatal presentation is considered to be rare, and in a case series including 129 patients, the median age at diagnosis was one year (range 0.1-13 years) [5]. We report a rare presentation of SDS in a neonate with persistent conjugated hyperbilirubinemia progressing into acute liver failure along with haematological abnormalities.

## Case presentation

Case report

A five-day-old term neonate was admitted with early-onset sepsis, feed intolerance, and hypoglycaemia. He was born out of a third-degree consanguineous marriage, with a history of first-trimester abortion in the mother in her previous pregnancy. Family history was insignificant. In this pregnancy, antenatal history was not significant. The baby was delivered through a lower segment caesarean section following non-progression of labour with a birth weight of 1,800 g and not requiring resuscitation at birth. Feeding was initiated within the first hour of life. The baby had dull activity and was noted to have hypoglycaemia, requiring a maximum glucose infusion rate of 10 mg/kg/minute (6-12 mg/kg/minute) on the second day of life. He gradually developed feed intolerance with abdominal distension and non-bilious aspirates. Antibiotics were started. The septic screen was positive, and the critical samples (serum insulin, serum cortisol and serum beta-hydroxybutyrate) were normal. Severe thrombocytopenia with a platelet count of 8,000 mm^-3^ required single-donor platelet (SDP) transfusions.

Course in the hospital

**Figure 1:**
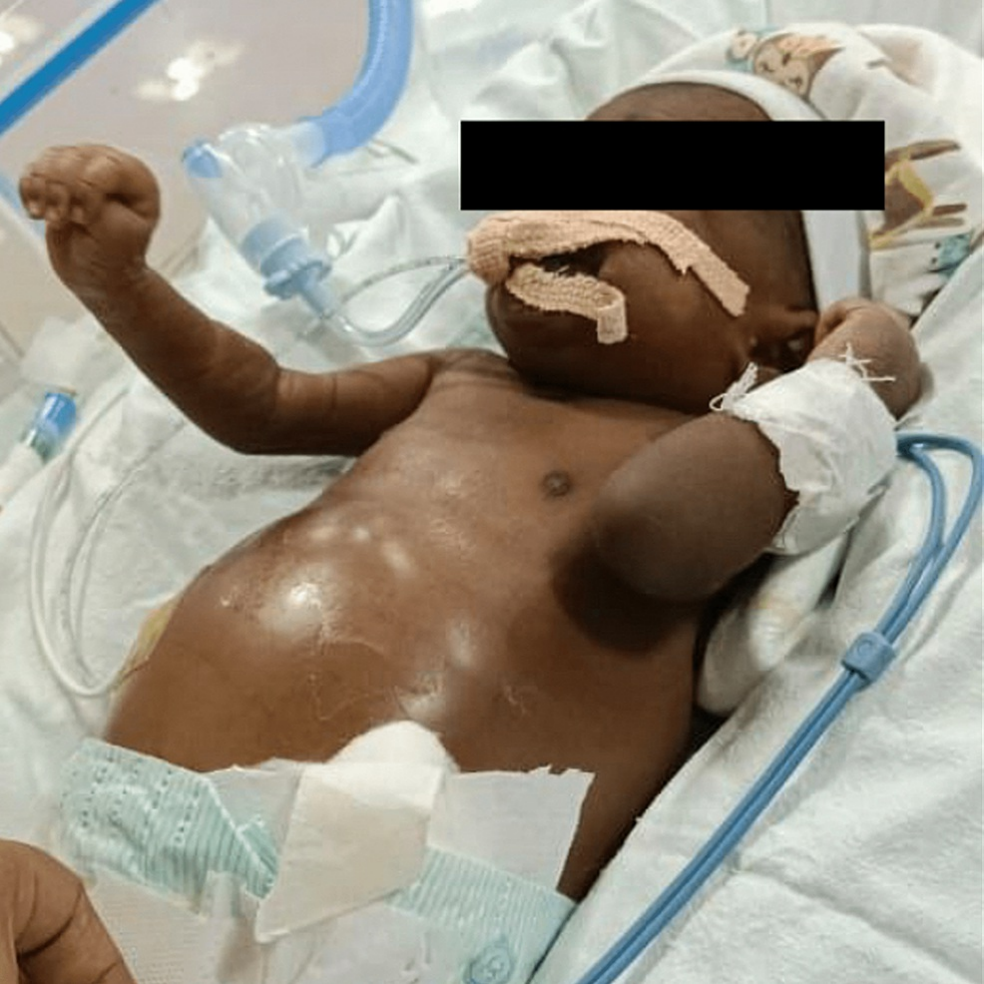
The baby on a ventilator with mild ascites on 20 days of life.

At admission day 5, the baby was euthermic with normal capillary filling time and stable vitals, with a pulse rate of 138 beats per minute and a respiratory rate of 44 beats per minute. The abdominal examination showed a soft, distended abdomen with hepatomegaly (liver span 8 cm). Bowel sounds were present, and orogastric tube aspirates were non-bilious. Given persistent thrombocytopenia, less than 30,000 mm^3^, a repeat dose of SDP was transfused, following which thrombocytopenia improved platelet count was 100,000 mm^-3^ (100,000-400,000 mm^-3^). The stool test was normal. The baby developed conjugated hyperbilirubinemia, with the total serum bilirubin (TSB) being 14.46 mg/dL (0-12 mg/dL) and a direct fraction of 7.6 mg/dL (<2 mg/dL). Liver enzymes were normal. The total serum protein level decreased. The blood culture was reported as sterile. The thyroid profile was normal with free T_4_ 1.9 ng/dL (0.9-3.4 ng/dL) and thyroid stimulating hormone (TSH) 6 mU/L (1.2-13.1 mU/L). The ultrasound of the abdomen showed mild ascites with sluggish peristalsis. With a provisional diagnosis of neonatal cholestasis secondary to sepsis, antibiotics were continued and vitamin supplements were started. On day 9, liver function tests showed significantly raised bilirubin levels of 32.8 mg/dL (TSB 0-15 mg/dL), with a direct fraction of 15.84 dL (<2 mg/dL) and normal liver enzymes. The coagulation profile was severely deranged: prothrombin time, or PT, 55 seconds (11-14 seconds); activated partial thromboplastin time, or APTT, 100 seconds (23-45 seconds); international normalised ratio, or INR, 5.2 (1.1-1.7), thereby requiring fresh frozen plasma transfusions. Ferritin was normal. The galactosemia panel was negative. An exchange transfusion was planned given the high TSB. A complete haemogram revealed macrocytic anaemia mean corpuscular volume (MCV) of 112 fL (95-105 fL), haemoglobin (Hb) of 9.6 gm/dL (12-15 mg/dL) and severe thrombocytopenia with a platelet count of 18,000 mm^-3^ (100,000-500,000 mm^-3^). Platelet transfusions were continued. A repeat blood culture grew *Escherichia coli*, and sensitive antibiotics were added. Tandem mass spectrometry (TMS) and urine gas chromatography-mass spectrometry (GC-MS) were negative. Repeat liver function tests and a coagulation profile showed a decreasing trend, falling to near-normal levels. However, the baby had persistent bi-cytopenia (anaemia and thrombocytopenia), for which multiple packed-cell RBC and platelet transfusions were administered. The bone marrow aspiration was inconclusive. By day 15, the abdominal distension had gradually progressed, causing respiratory distress and requiring ventilatory support. Ultrasound of the abdomen revealed gross ascites with the right perinephric collection. Diagnostic and therapeutic paracentesis was performed. Ascitic fluid analysis revealed a cell count of 70 cells mm^-3^ (<250 cells mm^-3^; neutrophils, 60%; and lymphocytes, 40%), protein of 694 mg% (<250 mg%), glucose of 97 mg% (<50 mg%) and a sterile culture. Repeated therapeutic paracentesis was required for this neonate (Figure 1).

After four days, liver function tests started to rise again, showing conjugated hyperbilirubinemia with normal liver enzymes. The baby started to develop bilateral pitting oedema in the lower limbs, followed by anasarca. The baby soon developed encephalopathy and disseminated intravascular coagulopathy (DIC). Given persistent conjugated hyperbilirubinemia with features of liver failure, a liver biopsy was planned on day 24, which showed cholestasis with chronic hepatitis and macronodular cirrhosis. The baby clinically deteriorated with persistent refractory bi-cytopenia and significant hyperbilirubinemia, requiring multiple blood product transfusions. Whole exome sequencing was planned for day 27. The baby succumbed on day 31, with fulminant liver failure. A genetic report revealed a pathogenic variant of the SBDS gene with a homozygous mutation revealing SDS-1 (OMIM#260400). Inheritance is autosomal recessive. Both parents were heterozygous for the same mutation. The parents gave their consent. No ethical issues were involved. The laboratory's values about the measured and reference values are shown in Table 1.

**Table 1:** Laboratory results reference values and measured values. TSH, thyroid stimulating hormone; PT, prothrombin time; APTT, activated partial thromboplastin time; INR, international normalised ratio; Hb, haemoglobin; MCV, mean corpuscular volume

Laboratory parameter	Reference values	Measured values
Platelet count on day 5	100,000-400,000 mm^-3^	30,000 mm^-3^
Platelet count on day 9	100,000-400,000 mm^-3^	18,000 mm^-3^
Total bilirubin on day 5	0-12 mg/dL	14.46 mg/dL
Conjugated bilirubin on day 5	<2 mg/dL	7.6 mg/dL
Free T4	0.9-3.4 ng/dL	1.9 ng/dL
TSH	1.2-13.1 mU/L	6 mU/L
Total bilirubin on day 9	0-15 mg/dL	32.8 mg/dL
Conjugated bilirubin on day 9	<2 mg/dL	15.84 mg/dL
PT	55 seconds	55 seconds
APTT	23-45 seconds	100 seconds
INR	1.1-1.7	5.2
Hb	12-15 mg/dL	9.6 mg/dL
MCV	95-105 fL	112 fL
Ascitic fluid cells	<250 cells mm^-3^	70 cells mm^-3^
Ascitic fluid protein	<250 mg%	694 mg%
Ascitic fluid sugars	<50 mg%	97 mg%

## Discussion

SDS is an autosomal recessive inherited disorder primarily affecting the pancreas and bone marrow. Clinical manifestations include a triad of exocrine pancreatic insufficiency, haematological dysfunction and skeletal abnormalities. It more commonly affects males, with the age of presentation being infancy. The earliest manifestation in infancy is pancytopenia, especially neutropenia [6]. The classic presentation of SDS in infancy or early childhood is failure to thrive, growth retardation, steatorrhoea, recurrent infections, bone marrow failure and aplastic anaemia [7]. At presentation, nearly all affected children have intermittent or persistent neutropenia [8]. Skeletal abnormalities include osteopenia, short stature, asphyxiating thoracic dystrophy due to rib cage restriction and severe spondylometaphyseal chondrodysplasia [9]. SDS is a multiorgan genetic disorder, with a varied spectrum of clinical manifestations ranging from the typical triad to uncommon or rare presentations. Some would include single features of the triad and more subtle or clinically inapparent findings where genetic testing for SBDS gene mutations would play a key role in diagnosis. Uncommon manifestations include cardiac abnormalities, liver dysfunction, neurocognitive and behavioural problems, immunological dysfunction, myelodysplasia and acute myeloid leukaemia, dental dysplasia, endocrine abnormalities, non-haematopoietic malignancies, etc. [4].

We report a male neonate born out of third-degree consanguineous marriage presenting with feed intolerance, hypoglycaemia, sepsis, thrombocytopenia and conjugated hyperbilirubinemia. The baby gradually developed persistent bi-cytopenia (macrocytic anaemia and thrombocytopenia), requiring multiple transfusions and progressive liver failure. Hepatic involvement in SDS is rare, and if it occurs, it has a milder presentation that includes elevated transaminases and a fatty liver. This is usually transient and resolves over time [4]. SDS with abnormal transaminase levels was detected in neonates, and these returned to normal with increasing age [6]. However, in our case, the severity of hepatic involvement in a neonate to the extent of cirrhosis and liver failure is very unusual. Hepatic involvement in SDS is most commonly associated with pancreatic dysfunction in the form of steatorrhoea, malnutrition and failure to thrive, along with a universal finding of haematological abnormality [6,10].

This case highlights the isolated hepatic presentation of SDS along with persistent bi-cytopenia in a neonate. Histopathological abnormalities on liver biopsy include varying degrees and combinations of steatosis, cellular inflammation and fibrosis [11]. Cirrhosis has rarely been reported in association with SDS, with no reports in over 50 years [12]. In our case, a liver biopsy was suggestive of cholestasis with chronic hepatitis and macronodular cirrhosis. Cirrhosis and liver failure, a rarely reported complication, are most commonly seen beyond infancy, unlike in our case. In a review by Bodian et al., five children were described with SDS and cirrhosis, which was discovered at autopsy in all cases [13]. Out of the five, the youngest child was only two years old and was described as having *early cirrhosis*. Camacho et al. described cirrhosis in SDS in a 16-month-old child and described it as *extremely early cirrhosis *[10]. We reported a neonate with cirrhosis and liver failure, thus describing our case as having *extremely early neonatal cirrhosis*. In our case, the diagnosis was established using whole exome sequencing, which revealed a pathogenic variant of the SBDS gene with a homozygous mutation revealing SDS-1 (OMIM#260400). Thus, inheritance is autosomal recessive.

The SBDS gene codes for the SBDS protein. The protein is found predominantly in the nucleotide and is implicated in the nucleolus and involved in the normal functioning of the ribosome, amplification of the centrosomes and leukaemogenesis. However, the true function of proteins is unknown. Our case would have required liver transplantation due to cirrhosis and also haemopoietic cell transplantation (HCT) due to persistent and refractory bi-cytopenia. However, our patient gradually deteriorated and succumbed owing to liver failure.

## Conclusions

With this case report, we'd like to highlight an incredibly uncommon SDS manifestation in the form of extremely early neonatal cirrhosis. We are unaware of any reports, to our knowledge, of newborn presentations of hepatic cirrhosis and full-blown liver failure in SDS combined with prolonged refractory bi-cytopenia. Genetic investigations are crucial for making a diagnosis in these uncommon presentations and allowing parents to receive genetic counselling as well as establishing future management plans.
